# Psychological characteristics of Japanese patients with chronic pain assessed by the Rorschach test

**DOI:** 10.1186/1751-0759-4-20

**Published:** 2010-11-26

**Authors:** Kazumi Yamamoto, Kenji Kanbara, Hiromi Mutsuura, Ikumi Ban, Yasuyuki Mizuno, Tetsuya Abe, Maki Yoshino, Aran Tajika, Yoshihide Nakai, Mikihiko Fukunaga

**Affiliations:** 1 Department of Psychosomatic Medicine, Kansai Medical University, Osaka, Japan; 2 Department of Psychosomatic Medicine, Rakusai Newtown Hospital, Kyoto, Japan; 3 Department of Neuropsychiatry, Kansai Medical University, Osaka, Japan

## Abstract

**Background:**

The increasing number of patients with chronic pain in Japan has become a major issue in terms of the patient's quality of life, medical costs, and related social problems. Pain is a multi-dimensional experience with physiological, affective, cognitive, behavioral and social components, and recommended to be managed via a combination of bio-psycho-social aspects. However, a biomedical approach is still the dominant method of pain treatment in Japan. The current study aimed to evaluate comprehensive psychological functions and processes in Japanese chronic pain patients.

**Methods:**

The Rorschach Comprehensive System was administered to 49 in-patients with non-malignant chronic pain. Major variables and frequencies from the test were then compared to normative data from non-patient Japanese adults by way of the t-test and chi-square test.

**Results:**

Patients exhibited high levels of emotional distress with a sense of helplessness with regard to situational stress, confusion, and ambivalent feelings. These emotions were managed by the patients in an inappropriate manner. Cognitive functions resulted in moderate dysfunction in all stages. Information processing tended to focus upon minute features in an inflexible manner. Mediational dysfunction was likely to occur with unstable affective conditions. Ideation was marked by pessimistic and less effective thinking. Since patients exhibited negative self-perception, their interpersonal relationship skills tended to be ineffective. Originally, our patients displayed average psychological resources for control, stress tolerance, and social skills for interpersonal relationships. However, patient coping styles were either situation- or emotion-dependent, and patients were more likely to exhibit emotional instability influenced by external stimuli, resulting in increased vulnerability to pain.

**Conclusions:**

Data gathered from the Rorschach test suggested psychological approaches to support chronic pain patients that are likely to be highly beneficial, and we thus recommend their incorporation into the course of current pain treatments.

## Background

The number of patients with chronic pain in Japan is estimated to be approximately 17 million [1]. Issues concerning the treatment of chronic pain, such as elevating medical expenses, inappropriate treatment measures and deteriorating quality of life for both patients and their families have resulted in immeasurable social loss, accompanied by personal and social factors associated with the aging society [2,3].

Pain of any kind can be a strong impetus for patients to seek medical care as they assume that pain indicates a serious medical matter. Pain is defined by the International Association for the Study of Pain (IASP) as "an unpleasant sensory and emotional experience associated with actual or potential tissue damage, or described in terms of such damage" and regarded as chronic when non-malignant pain persists for more than six months [4]. Emotional distress related to pain is perhaps the most negative aspect, in addition to physical pain, and must be considered as a multi-dimensional experience that has physiological, affective, cognitive, behavioral and social components for treatment [5]. In accordance with the multitude of factors associated with pain, a multidisciplinary approach was developed [6] and demonstrated to be the most efficacious and cost-effective treatment for chronic pain [7-9]. Few practitioners in Japan, however, have adopted this approach, as several issues complicate its implementation, such as conflicts in establishing a multidisciplinary team, under-developed treatment programs, and insufficient medical funding. Under such circumstances a conventional biomedical approach is likely to be effected or even reinforced [10,11].

Most of our chronic pain patients appeared to be exhausted both physically and psychologically when referred to our Department of Psychosomatic Medicine. Patients often complained of dissatisfaction with previous treatments and distrust of medical professionals. Consequently, our physicians often experienced difficulty in establishing therapeutic relationships with these patients at the time of admission. Kenny suggests that a fundamentally complicating factor between a patient and his/her physician may originate from "struggles for legitimating the cause of pain", as either biogenic or psychogenic [12]. This is particularly important in conventional biomedical settings, where the issue of whether pain is "real or not" is crucial for treatment. Furthermore, patients and doctors challenge each other's credibility in such cases, which undermines the quality of their interactions and causes each other distress [12]. When 85% of lower back pain cases have no physical basis that can be identified [13], patients with medically unexplained disorders may have negative experiences during medical encounters. This type of doctor-patient relationship is not therapeutic and causes an additional source of stress[12,14]. It would be of great help for medical professionals to consider psychological aspects of pain treatment for patients with chronic pain, in order to ensure that pain management is both beneficial and therapeutic.

Psychological aspects of chronic pain patients have been the subject of many studies, which have revealed important psychological constructs that can prolong or exacerbate pain, such as pain catastrophizing [15], fear avoidance [16], low self-efficacy [17] and coping strategies [18]. Not all of these pain-related assessment measures are available in Japan, although several studies have examined personality and mood status of chronic pain patients by use of measures such as the Cornell Medical Index (CMI), the Self-rating Depression Scale (SDS), the Manifest Anxiety Scale (MAS), the Tokyo University Egogram (TEG), and the Minnesota Multiphasic Personality Inventory (MMPI). These findings indicated that the psychological features experienced by chronic pain patients are neurotic, depressive and hypochondriac in nature. Patients also exhibit defensive tendencies. These features result from chronic pain and indicate that psychological aspects are necessary in the treatment regardless of the cause of pain [19,20].

Psychological measures of these studies were obtained by self-reporting questionnaires which focused mainly upon the assessment of psychopathology. However, findings often appeared to be due to the presence of chronic pain and related symptoms rather than psychopathology, and did not indicate the original psychological resources of the patients concerned. In consideration of the complexities of pain experiences, the Rorschach Comprehensive System (CS) could be used to provide multi-dimensional psychological aspects of chronic pain patients. This system investigates both original psychological resources and processes that generate symptoms and/or behaviors as they are similar to those in producing Rorschach responses. Furthermore, respondents' efforts to minimize problems might not bias the results as they are rarely aware of the type of interpretation to be made for their responses [21]. Using the Rorschach CS, Acklin and Bernat examined chronic low back pain (LBP) patients to address the association between LBP, depression, and alexithymia. These authors found Depression Index (DEPI) to be reduced in LBP patients along with Rorschach features consistent with alexithymia, and a number of similarities to the personality disorders group [22]. Alexithymia, conceptualized by Sifneos, describes a trait syndrome associated with difficulty identifying and communicating emotions, confusion between emotional and somatic sensations, and impoverishment of fantasy and capacity for symbolic thought [23].

The Rorschach CS has never been fully explored in Japanese patients suffering from chronic pain, and this study therefore aimed to investigate comprehensive psychological characteristics of Japanese patients with chronic pain.

## Methods

### Participants

One hundred and three (103) in-patients reporting non-malignant pain for more than six months were admitted to the Department of Psychosomatic Medicine in Kansai Medical University Hospital between January 2006 and June 2008. Twenty-three patients (22%) who were under 20 or over 70 years of age were excluded in order to compare the Rorschach data against control non-patient Japanese adults within the same age range. Nineteen patients (18%) could not be contacted upon their admission for logistical reasons, and ten (10%) were excluded due to physical conditions or as a result of decisions made by their attending physicians. Two patients (2%) refused to participate. After written informed consent had been obtained from the remaining forty nine (49) patients, the Rorschach CS was administered by the first author, a clinical psychologist. One patient was subsequently eliminated due to an insufficient number of responses, as normal protocol tests with less than 14 responses are considered invalid in the CS system [21], leaving forty eight (48) patients for data analysis.

Table 1 lists the breakdown of demographic variables and pain status. Mean patient age was 43.42 (±14.52, 21-68 yrs) with 14 males (41.07 ± 14.25) and 34 females (44.38 ± 14.72). The number of patients referred from other departments or hospitals was 44 (91.7%) and 4 patients visited the Department of their own accord (8.3%). The average number of hospitals visited prior to admission in our department was 5.13 (±2.75), with a range of 2 to 15. The median for pain duration before taking the Rorschach test was 4.19 years (±3.72 SD), with a range of 0.6 to 16.8 years. Fifteen patients (31%) exhibited mild depressive states, but none of our patients suffered from neurological problems or psychotic symptoms including major depressive episodes according to the DSM-IV criteria [24].

**Table 1:** Demographic and pain characteristics (n = 48)

Characteristics	Category	Frequency	Percentage (%)
Gender	Male	14	29.2
	Female	34	70.8

	Single	16	33.3
	Married	25	52.1
Marital Status	Divorced	4	8.3
	Unmarried-partner household	3	6.3

	<12 years	2	4.2
	12 years	20	41.7
Education Level	13-15 years	15	31.3
	15 < years	11	22.9

	Full-time	3	6.3
	Part-time	1	2.1
	Leave of absence	13	27.1
Employment	Retired	8	16.7
	Unemployed	10	20.8
	Housewife	11	22.9
	Student	2	4.2

	Medical treatment	9	18.8
	Stress	9	18.8
	Illness	7	14.6
Causes of Pain	Traffic accident	8	16.7
	Work-related injuries	5	10.4
	No identifiable	10	20.8

	Head, face and mouth	5	7.8
	Cervical region	5	7.8
	Upper shoulder and upper limbs	6	9.4
Regions of Pain (incl. multiple sites)	Thoracic region	1	1.5
	Abdominal region	5	7.8
	Lower back, lumber spine	23	35.9
	Lower limbs	6	9.4
	Pelvic region	2	3.1

### Psychological measures

The Rorschach Comprehensive System (CS) has standard administration rules and is the most commonly-used scoring system in the world. It has good inter-rater and test-retest reliability with good statistical construct validity [21,25]. In this system, seven major groups of variables, collectively referred to as a 'cluster', are evaluated: 1) control and stress tolerance, 2) information processing, 3) mediation, 4) ideation, 5) affect, 6) self-perception, and 7) interpersonal perception and behavior. It also provides data and reference samples for non-patient controls and for patients within the expected normative range for each variable, which then facilitates interpretation by using the standard deviation formula. Standard deviation indicates what is or is not within the normative limits used as the basis for the predictions.

### Scoring procedures

The responses of our chronic pain patients were scored by the first author, a clinical psychologist. Scorings were reviewed by a second clinical psychologist affiliated with a different department and finalized after discrepancies had been discussed. Further consultations were made with a Rorschach expert to finalize data when agreement was not reached between the two local psychologists. Both clinical psychologists were trained in the administration and scoring of the Rorschach CS and have extensive experience with this system.

### Statistical analysis

Means and frequency data of all major variables arising from the Rorschach CS were compared between chronic pain patients (hereafter referred to as 'CP patients') and non-patient adults (hereafter referred to as the 'NA group'). The latter group consisted of 200 males (35.26 ± 12.28) and 200 females (35.92 ± 12.02) with the mean age of 35.59 (±12.84) [26]. In terms of age and education, no significant difference was detected in the demographic data when compared between CP and NA groups. Significant differences were found, however, when performing a Student's t-test, or Welch's t-test when homogeneity of variance was not hypothesized. Frequency data for major variables or indices was examined using the chi-squared test. These were analyzed using SPSS v.11.5. For all analyses, a probability value of p < .05 was considered significant and variables were noted when they deviated from the expected value or range.

### Ethics

All procedures were submitted and approved by the Ethical Committee of Kansai Medical University Hospital.

## Results

Tables 2 and 3 list the major Rorschach variables for CP patients and for the NA group in terms of: 1) control and stress tolerance, 2) affect, 3) information processing, 4) mediation, 5) ideation, 6) self-perception, and 7) interpersonal perception and behavior. Some variables were plotted in more than one cluster of variables because they related to more than one characteristic. Further descriptions of the technical terms associated with the Rorschach test, their abbreviated form, and their interpretation, are discussed by Exner [21] and Takahashi [26].

**Table 2:** Comparisons of means of major Rorschach Variables

Cluster	Variable Name	Description	Characteristics Measured	CP (n = 48)		NA (n =400)		
					
				Mean	SD	Mean	SD	Sig.*
	AdiD	Adjusted D Score	capacity for control	-0.13	1.39	0.03	1.35	n.s.
	D	D Score	stress to lerance and elements of control	-0.62	1.54	-0.15	1.4	n.s.
Controls and stress tolerance	EA	Expreience Actual: M + WSumC	available resources	7.79	4.16	7.14	3.40	n.s.
	M	Human Movement Response	ability to fantasize	3.88	2.77	3.98	2.45	n.s.
	FM	Animal Movement Response	Peripheral thought by need experiences	3.77	2.85	3.67	2.39	n.s.
	m	Inanimate Movement Response	peripheral thought by external demand situations	1.40	1.32	1.13	1.15	n.s.
	Es	Experienced Stimulation: FM + Sum Shading	current stimulus demands	9.67	5.90	7.68	4.02	*

	L	Lambda	economizing use of resources	0.64	0.46	0.96	0.88	**
	Sum Shading	Sum of (C'+T+V+Y)	unusual distress experiences	4.50	4.04	2.88	2.21	**
	Sum C'	Achromatic Color Response	suppression or constraint of emotion	2.21	2.08	1.39	1.43	*
	Sum Y	Diffuse Shading Response	situational stress-related sense of helplessness	1.42	2.07	0.55	0.84	**
Affect	W Sum C	Sum Weighted Color	range of affective experiences	3.71	1.87	3.16	1.90	n.s.
	FC	Form-Color Response	well controlled or modulated emotional experiences	1.33	1.34	1.97	1.63	**
	CF	Color-Form Response	less restrained ventilation of feelings	2.67	1.84	1.98	1.57	*
	Blends	Multiple Determinants	cognitive complexity	4.13	3.38	2.98	2.18	*
	Col-ShdBlends	Color-Shading Blends	uncertainty, confusion ambivalence about feelings	0.92	1.11	0.34	0.62	**

	R	No. of Response	verbal productivity	22.02	8.45	23.51	6.90	n.s.
	W	Whole Response	commendable processing effort	10.73	4.93	11.53	4.59	n.s.
Information Processing	D	Common Details Response	less processing effort than W responses	7.25	5.59	9.55	5.65	**
	Dd	Unusual Detailos Response	more processing effort	3.81	4.44	2.44	1.97	*
	Zd	Processing Efficiency	efficiency of the scanning activity	0.22	5.80	-2.22	4.88	**
	PSV	Perseveration	problem in processing efficiency	0.63	1.14	0.31	0.61	*
	DQ+	Developmental Quality (+)	highest form of analysis and synthesis	6.48	3.47	5.88	3.01	n.s.

	XA%	Form Appropriate Extended	mediational activities for behaviours appropriate for situation	0.86	0.13	0.92	0.06	**
	WDA%	Form Appropriate-Common Areas	mediational activities for behaviors appropriate for obvious situation	0.88	0.12	0.93	0.06	*
	X-%	Distorted Form	disregard or distortion of reality, mediational dysfunction	0.16	0.17	0.08	0.06	**
Mediation	FQx-	Form Quality Minus Responses	distorted, arbitrary, unrealistic use of form	3.50	4.26	1.96	1.67	*
	S-	Minus Response in S Location	mediational dysfunction by negativisim or anger	0.75	1.16	0.37	0.65	*
	P	Popular Response	social conventionality	5.42	2.31	5.48	1.79	n.s.
	X+%	Conventional Form Use	common or conventional mediational decision	0.67	0.17	0.75	0.11	**
	Xu%	Unusual Form Use	less conventional and more idiographic	0.17	0.08	0.17	0.09	n.s.

	MOR	Morbid Content	pessimistic conceptual thinking	0.98	1.73	0.42	0.72	*
	Ma	Active Human Movement	positive conceptual ideation or behavior	2.69	2.24	2.23	1.91	n.s.
	Mp	Passive Human Movement	conceptual ideation of fantasy for defense from reality	1.35	1.90	1.75	1.48	n.s.
	Intell	Intellectualization Index	use of intellectualization as defensive tactic	1.35	1.67	1.57	1.61	n.s.
	Wsum6	Weighted Sum of 6 Special Scores	issue of ideational clarity	9.96	13.03	2.44	3.48	**
Ideation	DV	Deviant Verbalization	distorted language use or idiosyncratic modes of expression	0.54	0.97	0.14	0.38	**
	DR	Deviant Response	indecisiveness or a defensive attempt to detach from the task at hand	1.33	2.04	0.06	0.23	**
	INCOM	Incongruous Combinations	conceptual failure to discriminate and/or a kind of concrete reasoning	0.04	0.20	0.23	0.53	**
	FAB	Fabulized Combinations	less mature forms of ideation	0.69	1.24	0.40	0.74	n.s.
	ALOG	Inappropriate Logic	poor judgement influencing conceptualization	0.42	1.18	0.01	0.11	*
	CONTAM	Contamination	the most severe ideational disorganization	0.00	0.00	0.00	0.00	n.s.

	Fr+rF	Reflection Responses	narcissistic-like feature of personality	0.23	0.69	0.20	0.53	n.s.
	3r+(2)/R	Egocentricity Index	self-concern and self-esteem	0.31	0.31	0.31	0.14	n.s.
Self-perception	FD	Form Dimension Response	ability for introspection	0.60	0.82	0.61	0.78	n.s.
	Sum V	Shading-Dimensionality Response	chronic preoccupation with negative features of the self	0.65	1.08	0.34	0.62	*
	MOR	Morbid Content	negative or self-image	0.98	1.73	0.42	0.72	*

	Sum T	Texture Variable	openness to close emotional relations	0.23	0.69	0.20	0.53	n.s.
	Human Cont	Number of Human Response	interpersonal interest	5.63	4.98	5.67	3.09	n.s.
Interpersonal perception and behavior	H	Whole Real Human	interpersonal interest and empathy	2.54	2.14	3.09	1.92	n.s.
	GHR	Good Human Representational Responses	effective and adaptive interpersonal behaviors	3.52	2.11	4.30	2.33	*
	PHR	Poor Human Representational Responses	ineffective or maladaptive interpersonal behaviors	3.06	4.15	1.97	1.71	*
	COP	Cooperative Movement	positive interpersonal exchanges	1.02	1.26	1.27	1.27	n.s.
	AG	Aggressive Movement	aggressive or competitive interpersonal exchanges	0.83	1.08	0.34	0.66	**
	PER	Personal Response	intellectual authoritarianism or defensiveness	1.19	1.54	0.28	0.66	**

Sig.* Significance, **p < .01, *p < .05, n.s. no significant difference CP: Japanese chronic pain patients, NA: non-patient adults

**Table 3:** Frequencies and percentages of major Rorschach variables

Cluster	Variables	CP (n = 48)		NA (n = 400)		P value	
		No.	%	No.	%		
	Introversive	9	19	132	33	0.045	*
Controls and stress tolerance	Ambitent	27	56	211	53	0.646	n.s.
	Extratensive	12	25	57	14	0.051	†

	S-CON positive	3	6	2	1	0.000	**
Affect	DEPI >4	17	35	80	20	0.014	*
	FM + m < Sum Shading	17	35	78	20	0.011	*
	(CF + C) > FC + 2	17	35	48	12	0.000	**

	HVI positive	10	21	46	12	0.065	n.s.
	Zd > +3.0	12	25	56	14	0.045	*
	Zd < -3.0	12	25	163	41	0.035	*
	PTI >3	1	2	0	0	0.004	**
Cognition: input, mediation, ideation	XA% > 0.89	20	42	260	65	0.002	**
	XA% < 0.70	5	10	1	0	0.000	**
	WDA% < 0.75	3	6	3	1	0.002	**
	X-% > 0.30	6	13	1	0	0.000	**
	X+% < 0.55	9	19	10	3	0.000	**
	MOR > 2	6	13	9	2	0.000	**
	Mp > Ma	8	17	139	35	0.012	*

Self-perception and Interpersonal relationship	CDI >3	12	25	116	29	0.562	n.s.
	Sum T = 0	39	81	228	57	0.001	**
	Pure H = 0	7	15	17	4	0.003	**
	AG > 2	3	6	1	1	0.000	**

**p < .01, *p < .05, +p < .10, n.s. no significant difference

S-CON (the Suicide Constellation: self-destructive behavior), DEPI (the Depression Index: depressive mood), HVI (the Hypervigilance Index: hyperalertness), PTI (the Perceptual-Thinking Index: cognitive dysfunction), CDI (the Coping Deficit Index: social immaturity)

### Control and stress tolerance (Tables 2 &3)

Here, we examined the capacity to make decisions and implement specific behaviors that meet the demands of situations which involve the use of resources, stimulus demands, and stress tolerance. The Adjusted D score (AdjD, one's capacity for control), the D score (D, stress tolerance and elements of control), and the Experience Actual score (EA, available resources to make it possible to adjust one's own needs and emotions to match external reality) in CP patients were within the expected range, and no significant inter-group differences were apparent. The Experienced Stimulation score (es, stimulus demands) of CP patients was within the expected range, but was significantly higher than that of the NA group (p < .05, Table 2). Three types of coping styles have been identified: (i) introversive--introspection-based; (ii) ambitent--inconsistent or flexible in using emotions or introspection according to situations; and (iii) extratensive--emotion-based. The frequency of introversive style in CP patients was significantly lower (19%) than the NA group (33%; p < .05, Table 3). No inter-group difference was found in the frequency of ambitent style between CP patients (56%) and the NA group (53%). The frequency of extratensive style in CP patients was relatively higher (25%) than the NA group (14%; p < .10, Table 3).

Control and stress tolerance data suggest that CP patients have originally adequate capacities for control and stress tolerance and utilize appropriate psychological resources as with most adults (EA, AdjD and D). Fewer CP patients exhibited the introversive coping style, implying that fewer CP patients reasoned things through while keeping emotions aside before making decisions or problem-solving. Half of CP patients and half of the NA group were classified as exhibiting the ambitent style, which is an inconsistent or flexible way of using thoughts and emotions according to situations. More CP patients exhibited the extratensive style, suggesting that they tend to invest more of their feelings into decision-making and/or problem-solving processes and are more likely to use interactions with their environment as a source of information and/or gratification.

### Affect (Tables 2 &3)

Variables relating to affect examine the role of emotions in the psychological function and organization of the person. The frequency of a positive Suicide Constellation (S-CON, self-destructive preoccupation) in CP patients was significantly higher (6%) than the NA group (1%; p < .01, Table 3). Lambda (L, economic use of resources) was significantly lower in CP patients than the NA group (p < .01, Table 2). The frequency of a positive depression index (DEPI > 4, an implicit depressive mood) in CP patients was significantly higher (35%) than the NA group (20%; p < .05, Table 3). SumShading score (sum of C' + V' + T + Y, unusual distress experiences) in CP patients revealed higher deviation from the expected range, and was significantly higher than the NA group (p < .01, Table 2). The frequency of FM + m < SumShading, more distress or emotional discomfort, in CP patients was significantly higher (35%) than the NA group (20%; p < .05, Table 3). The Achromatic Color variable (SumC', excessive internalization of feelings) in CP patients revealed higher deviation from the expected range, and was significantly higher than that of the NA group (p < .05, Table 2). The Diffuse Shading variable (SumY, situational stress-related psychological helplessness) in CP patients revealed higher deviation from the expected value, and was significantly higher than the NA group (p < .01, Table 2). Multiple determinants (Blends, psychological complexity) in CP patients revealed higher deviation from the expected value, and was significantly higher than the NA group (p < .05, Table 2). The Color-Shading blends (Col-Shading, confusion or ambivalence of feelings) in CP patients revealed slightly higher deviation from the expected range, and was significantly higher than the NA group (p < .01, Table 2). With regard to the modulation of emotional discharge, the Form Color response (FC, well-controlled emotional experiences with situation-appropriate expressions) in CP patients was significantly lower than the NA group (p < .01, Table 2); whereas the Color Form responses (CF, less restrained forms of affective discharge/expression) in CP patients was significantly greater than the NA group (p < .05, Table 2). The frequency of (CF + C) > FC + 2 in CP patients was significantly higher (35%) than the NA group (12%; p < .01, Table 3).

In summary, the data relating to affect suggest that CP patients experienced unusually high levels of distress and/or emotional discomfort (FM + m < SumShading), such as self-destructive thoughts (S-CON), depressive mood (DEPI > 4) and a sense of helplessness due to situational stress (SumY). These patients did not use the tactic of psychologically ignoring the complexity and/or ambiguity of a field (L), and their psychological function and processing seemed more complicated and confused by ambivalent feelings (Blends, Col-Shading). Their modulation of emotional discharge was likely to be unstable; in other words, CP patients exhibited a tendency to either excessively internalize their feelings (SumC'), or discharge them expulsively in a more uncontrolled manner (FC, CF, and CF + C > FC + 2).

### Cognitive functions (Table 2 &3)

Here, we examined three aspects of cognitive function, or a cognitive triad, i.e. (i) information processing; (ii) cognitive mediation; and (iii) ideation, thinking process leading to some form of mental conceptualization of translated information.

Variables of information processing assess mental procedures entailed in the input of information. There were no significant differences in the total number of responses (R) to 10 inkblots between CP patients (22.02 ± 8.45) and the NA group (23.51 ± 6.9), or when considering the Whole response (W, commendable processing effort) and the Hypervigilance Index (HVI, hyperalertness). Common Detail response (D, easy or economical scanning) was significantly lower in CP patients than the NA group (p < .01, Table 2). Unusual Detail response (Dd, focus more on minute or unusual features of a new field of information with more processing effort) in CP patients deviated significantly from the expected range, and was significantly higher than the NA group (p < .05, Table 2). The Zd value (Zd, efficiency of scanning activity during information processing) was significantly higher in CP patients than the NA group (p < .01, Table 2). Perseverations (PSV, difficulty in shifting attention) was slightly higher than expected, and was significantly higher in CP patients than the NA group (p < .05, Table 2). No significant inter-group differences were found in terms of Developmental Quality (DQ, quality of processing activity).

In summary, information processing data suggest that CP patients are less likely to use economical scanning to gain new information (D); instead, they focus more on minute or unusual features within a new field of information (Dd). Scanning efficiency and the quality of processing activity of CP patients appears more than adequate compared to the NA group (Zd), but more patients in the CP patient group exhibited a little difficulty in shifting their attention (PSV).

Variables concerning cognitive mediation assess mental operations that translate or identify inputted information. The Perceptual-Thinking Index positive score (PTI > 3, mediational and ideational difficulties) was significantly greater in CP patients than the NA group (p < .01, Table 3). XA% (appropriate form use) and WDA% (appropriate form use in common areas) in CP patients was within the expected range, but was significantly lower than the NA group (p < .01, p < .05 respectively, Table 2). Form Quality minus responses (X-% & FQ-, distorted form use) deviated significantly from the expected range in CP patients, and was significantly greater than the NA group (p < .01, p < .05 respectively, Table 2). Distorted Space response (S-, mediational dysfunction due to negativism or anger) in CP patients was slightly higher than expected, and was significantly higher than the NA group (p < .05, Table 2). Appropriate/common good form (X + %) in CP patients was within the expected range, but was significantly lower than the NA group (p < .01, Table 2). Less conventional and more idiographic form (Xu%) and Popular response (P, expected or acceptable responses) in CP patients were within the expected range, and no inter-group significant differences were evident.

In summary, cognitive mediation data suggest that with CP patients, mediation was usually appropriate for the situation, or that they exhibited the basic skills necessary to interact successfully with situations around them, although they were less appropriate than those of the NA group (XA%, WDA%). The probability of fewer conventional responses occurring in simple and/or precisely-defined situations with CP patients was low (Xu%, P), even if problems were observed (X + %). However, CP patients exhibited a moderate elevation in the incidence of mediational dysfunction (PTI > 3, X-%, FQ-), when associated directly to unstable affective conditions, particularly relating to possible feelings of negativity or anger (S-).

Variables concerning ideation assess conceptualization of translated inputs. Morbid content (MOR, pessimistic conceptual thinking) in CP patients showed a slightly higher incidence from the expected value, and was significantly greater than that in the NA group (p < .05, Table 2). The frequency of Mp > Ma (passive human movement > active human movement, tendency to defensively fantasize about reality) in CP patients was significantly lower (17%) than that in the NA group (35%; p < .05, Table 3). The Weighted sum of six special scores (Wsum6: DV, DR, INCOM, FABCOM, ALOG, and CONTAM; difficulties in conceptual thinking and issue of ideational clarity) of CP patients deviated widely from the expected ranges, and was significantly greater than that of the NA group (p < .01, Table 2). In terms of the Sum6 special scores, Deviant Verbalization (DV, distorted language use or idiosyncratic modes of expression) in CP patients showed a slightly higher incidence from the expected value, and was significantly higher than the NA group (p < .01, Table 2). Deviant Response (DR, indecisiveness or a defensive attempt to detach from the task at hand) in CP patients deviated greatly from the expected value, and was significantly higher than the NA group (p < .01, Table 2). Incongruous Combination (INCOM, conceptual failure to discriminate and/or form concrete reasoning) was significantly lower in CP patients than the NA group (p < .01, Table 2). Inappropriate logic (ALOG, strained or unconventional reasoning to justify the answer) in CP patients showed a slightly higher incidence from the expected value, and was significantly greater than the NA group (p < 0.5, Table 2).

In summary, ideation data suggest that CP patients' conceptual thinking was often distinguished by a moderately pessimistic mindset (MOR), but that they did not defensively substitute fantasy for reason in stressful situations (Mp < Ma). Furthermore, CP patients did not exhibit a conceptual failure with discrimination and/or the inability to use concrete reasoning (INCOM), but they did show cognitive mishaps (Wsum6) with regard to: 1) use of distorted language and/or idiosyncratic modes of expression (DV); 2) indecisiveness and/or a defensive attempt to detach themselves from the task at hand (DR); and/or 3) strained effort or use of unconventional reasoning to justify an answer (ALOG).

### Self- perceptions & Interpersonal-perceptions and behavior (Tables 2 &3)

Variables on self-perception assess self-image and self-involvement. Vista response (V, less positive introspective behavior) showed a slightly higher incidence from the expected value, and was significantly greater in CP patients than the NA group (p < .05, Table 2). Morbid content (MOR, negative self-image in self-perception) in CP patients showed a slightly higher incidence from the expected value, and was significantly greater than the NA group (p < .05, Table 2). The frequency of Pure H = 0 (less reality-based perception of self and others) was significantly greater in CP patients than the NA group (p < .01, Table 3).

In summary, self-perception data suggest that CP patients' introspective behavior on themselves tends to focus less on their positive sides (V), and that their self-image is likely to be negative (MOR). However, this perception of themselves or others might not always be based on reality (Pure H = 0).

Interpersonal perception and behavior data assess how a person perceives others, and how they will behave in various interpersonal situations. The frequency of positive Coping Deficit Index (CDI > 3, social immaturity or ineptness) showed no inter-group difference between CP patients and the NA group. Texture response (T, needs and openness to close emotional relations) was significantly lower in CP patients than the NA group (p < .01, Table 2). Good Human Representational response (GHR, good interpersonal behaviors and their effectiveness) was within the expected range, but was significantly lower in CP patients than the NA group (p < .05, Table 2). Poor Human Representational response (PHR, ineffective or maladaptive interpersonal behavior) was also within the expected range, but was significantly greater in CP patients than the NA group (p < .05, Table 2). Aggressive response (AG, aggressiveness or competitiveness on interpersonal exchanges) was slightly greater than the expected value, and significantly higher in CP patients than the NA group (p < .01, Table 2). Personal response (PER, way of reassuring oneself or warding off challenges from the examiner) was higher than the expected value and significantly greater in CP patients than the NA group (p < .01, Table 2).

In summary, interpersonal perception and behavior data indicate that CP patients are socially mature or developed, and that they have originally adequate interpersonal skills (CDI). However, they are generally not open to close emotional interactions, and can be apprehensive (T). They tended to perceive interpersonal exchanges as aggressive or competitive (AG) with less reality-based evidence (PureH = 0), and were warded off challenges (PER), which then led to less effective and/or maladaptive interpersonal behavior (GHR, PHR).

## Discussion

When pain becomes persistent, it often accompanies various types of aversive emotional and cognitive effects, which are likely to lead to deterioration of private and social lives. Rudy et al suggest that the characterization of pain as only a sensory discomfort of the pain-specific part of the body is not sufficient without inclusive perspective of emotional states and accompanying thoughts as the central role for clinically important pain control strategies [27]. The purpose of the present study was to assess comprehensive psychological functions or states of Japanese CP patients using the Rorschach CS. Our findings showed that patients originally had adequate psychological resources with capacities for control, stress tolerance (EA, AdjD) and adequate interpersonal skills (CDI). However, results also suggested that our CP patients exhibited some psychological issues including: 1) emotional issues, 2) cognitive dysfunction, and 3) defensive interpersonal relationships.

### Emotional issues

Affective states of CP patients revealed that they experienced highly unusual distress or discomfort (SumShading, FM + m < SumShading), including situational stress-related psychological helplessness (Y), self-destructive preoccupation (S-CON) and implicit depressive mood (DEPI). When we consider the IASP definition of pain, our present findings demonstrate that the pain experience is accompanied by various types of emotional distress in addition to pain specific discomfort [4]. Whether negative emotions are processed as causes or consequences of pain remains controversial, but can be clarified by conceptualizing both pain and emotion as multi-dimensional and sometimes overlapping processes with reciprocal influences upon each other [28]. All CP patients suffered from pain for more than six months. As for persistent pain, by examining functional relations between catastrophic thinking and the disability, Sullivan et al found that the chronicity of pain is an important moderator of psychological vulnerability for pain-related disability. The longer the duration of pain persists, an increasingly apparent sense of helplessness would make patients come to perceive themselves as being unable to manage pain effectively [29]. As shown by the significant value of Y in CP patients, persistent pain often disrupts customary private and social lives, and despondency and a sense of hopelessness become a likely outcome.

Perception of control has an important relationship with affective distress [30]. Patients who perceive their pain as an 'unexplainable mystery' may devalue their coping abilities and are less likely to rate their coping strategies as effective in controlling and reducing pain [31]. CP patients revealed significantly reduced proportions of introversive coping style, relatively high extratensive style, and almost identical ambitent style. These results suggested that more CP patients with an extratensive style tend to rely predominantly upon feelings or external feedback for reassurance rather than considering issues carefully before making decisions. Consequently, the impact of emotion is likely to make ideation more complex and illogical than the introversive style, which leads patients to trust their internal evaluations more than external feedback and to avoid being overly influenced by emotions [21]. Approximately half of patients suffering from CP exhibiting an ambitent style are flexible in using both thoughts and feelings simultaneously according to the situation [32]; however, with its less consistent and inefficient style, feelings may become overly influential in thinking, especially in an unfamiliar situation [21]. While any illness could make people feel vulnerable and less in control than usual, most CP patients exhibiting extratensive or ambitent coping styles are suggested to become susceptible to abnormal pain-related situations, thus rendering them emotionally vulnerable. This would make their coping styles more ineffective and result in a vicious circle.

Causal relationships between chronic pain and depression have been the subject of much debate. None of CP patients in the present study met the criteria for a major depressive episode, although DEPI positive frequency was significant compared to the NA group. Rudy et al found that when patients perceived a persistent pain as far beyond control, then they were more likely to increase affective distress, and proposed that the psychological mediators might be in the development of depression secondary to chronic pain [27]. A further research study by Ohayon et al found that a painful physical condition persisting for longer than 6 months could contribute to the prolongation of a depressive episode, and recommended evaluation of depression for patients with chronic pain [33].

As for emotional discharge, our CP patients showed excessive internalization or inhibition of emotions (C'), and were expressive in an uncontrolled manner (FC, CF, CF + C > FC + 2). Their emotions were not well controlled and expressed in extreme manners. This could be explained by the emotional states of CP patients showing significant low L, high Blends and Col-Shading, indicating that patients were suffering psychological complexity such as confusion and ambivalent feelings that they were not able to articulate properly. Also, emotions involving diffuse bodily states are often chaotic and change rapidly such that the language available to describe emotional states tends to be vague or amorphous [28]. The high value of C' is likely to occur in response to several factors. First, our CP patients were so confused by emotional distress that they preferred to avoid dealing directly with their feelings or that they did not trust their ability to control them. A second factor might be the patients' senses of insecurity about sharing their feelings with others or displaying them openly. However, the high incidence of C' is thought to relate to irritating feelings due to the inhibition or excessive internalization of emotion [21], and the affect is likely to bring about adverse health outcomes [34]. Furthermore, even suppression of unwanted thoughts or catastrophizing thoughts has been reported to heighten pain experiences [35,36]. These results suggest that consideration should be afforded to provide CP patients with the opportunity to be listened to and express their feelings regarding pain experiences in a safe and accepted relationship.

With regards to alexithymia, Acklin and Bernat proposed the trait for this condition in patients with low back pain based upon results from ambitent coping style and seven Rorschach constrictedness variables, including low R, low M, low WsumC, low FC, low Blends, high Lambda, and low EA [22]. The results of the present study did not fulfill the above condition except for high frequency of ambitent style and low FC, suggesting that our CP patients were not regarded as alexithymic, but further investigations are needed to be sure.

### Cognitive issues

Three cognitive functions, the 'Cognitive Triad' have been defined as: 1) information processing, 2) cognitive mediation, and 3) ideation. These are collective functions and reflect a continuous or circular process to form the basis of all deliberate and/or meaningful behaviors [21].

Information processing results demonstrated that our CP patients actively processed information effectively (D), and their scanning efficiency (Zd) and quality of processing activities (DQ +) were usually more adequate than the NA group. However, CP patients were likely to focus more upon minute or unusual features of a new field of information (Dd), and had a tendency to be inflexible in shifting their attention (PSV). This is reminiscent of the features of chronic pain patients who tend to be attentive to their pain and their complaints sometimes are felt as obstinate. Their focus on pain might lead to increased pain stimuli. Tracy et al reported that increased attention to pain increased pain intensity, whereas significant pain reduction was observed during distraction [37].

Cognitive mediations evaluate how the inputted image is identified or translated, focusing the extent to which the person acknowledges external reality when making decisions, as opposed to being influenced by psychological status. Cognitive mediations of our CP patients were less appropriate (XA%, WDA%) than the NA group, but the probability of a less conventional response (P, Xu%) occurring was minimal in simple and/or precisely defined situations. However, their mediation showed moderate dysfunction (X-%, FQ-, S-) with deviations from expected values. The significant incidence of these minus responses in CP patients indicated that some of their personal aspects caused the stimulus field to be disregarded and replaced by internal aspects projected into the response. Minus responses could be provoked by many reasons such as ideational sets, preoccupations and emotional elements, and all kinds of emotions can interfere with mediation [21]. As indicated by the high frequency of S-, some negative feelings such as anger were included and were likely to promote mediational dysfunction in CP patients.

Ideation focuses upon the characteristics of thinking, the quality and clarity of ideation, the frequency with which associated aspects manifest, and the manner in which it is used. Results from our CP patients suggested difficulties in conceptual thinking or an issue of ideational clarity as shown in the highly significant values of MOR and WSum6. MOR, a moderate pessimistic set, suggested that CP patients anticipate gloomy outcomes for their efforts, and that this negative perception is likely to be reinforced and interact with a sense of helplessness, as indicated by emotional issues. In addition, when the highly significant value of ALOG, which represents a tendency of unconventional or strained reasoning to justify the answer, is applied to pain and pain-related situations, it is likely to promote a type of catastrophic pain thinking and therefore contribute to a more intense pain experience and increased emotional distress [15]. This may also relate to negative expectations concerning the patients' own abilities which are likely to result in pain-related dysfunctional behaviors, such as fear of movement [38]. CP patients also showed significantly elevated values of DV and DR, distorted language use and indecisiveness or poor judgment, which often reflect a type of cognitive carelessness. These ideational dysfunctions suggest that our CP patients experienced some difficulty in formulating or expressing aspects of thinking in a less clear and less sophisticated manner, which is likely to impede their abilities to communicate clearly.

In a medical setting, the National Institute for Japanese Language found that most Japanese patients had the experience of being puzzled by unfamiliar words in conversations with physicians [39]. In doctor-patient inter-relationships, the ability to communicate effectively is crucial for patients to obtain the true benefits of medical advances and for physicians to achieve professional satisfaction [40]. In consideration of such cognitive dysfunctions in CP patients, as Pilowsky recommended, it would be helpful for them to be given clear explanations concerning the mechanisms and reactions of pain using readily understandable terminology and to provide ample opportunities for discussion and questions, which may need to be provided repeatedly [41].

### Interpersonal relationships

First, it is useful to point out that our CDI negative results (Table 3) suggest that CP patients were not socially immature and that they had adequate interpersonal skills. However, our self-perception findings suggest that CP patients focused on their less positive aspects (MOR, V). A significantly high value of Y, sense of helplessness, could contribute to lower self-efficacy, which is closely related to the sense of control over aversive stimulation. Arnstein et al identified self-efficacy as a significant predictor of the extent to which patients with chronic pain become disabled and/or depressed [17].

Our self-perception data contribute to our understanding of interpersonal perceptions and behaviors. Significantly low T and high PER values suggest that CP patients tended to be overly concerned with personal space and that they were much more cautious or defensive about creating or maintaining close emotional ties with others. Significantly high AG values showed that CP patients tended to perceive interpersonal exchanges as aggressive or competitive without reality-based evidence. Thus, their interpersonal behavior might not always be effective and adaptive. These findings indicate that CP patients appeared to lack emotionally close relationships and to be isolated. This situation is likely to make chronic pain patients focus more upon pain and their misery, as Turk and Flor found in many chronic pain patients whose preoccupation with their own bodies would lead to increased awareness and overestimation of sensory information [6].

Rorschach CS data compiled from our CP patients were examined in terms of emotional, cognitive and interpersonal aspects, as well as their original psychological resources. Findings suggest that pain and pain-related situations were mostly viewed as major stressors in patients' lives that triggered a certain degree of emotional distress, cognitive dysfunction and maladaptive interpersonal relationships. Our results did not indicate psychopathology, a finding consistent with previous studies by Gatchel & Okifuji and Maruta [7,42]. Physicians have been reported to show a tendency to prioritize the assessment of physical damage or disease over emotional reactions or the psychological wellbeing of patients with chronic pain [28]. However, persistent pain is highly disconcerting for patients with little knowledge about physical functions or psychological approaches. Consequently, consideration of psychological assessment and some intervention as part of the stress management of pain is likely to be helpful for patients with chronic pain in order to maintain their daily lives as much as possible, regardless of pain [43-45].

Our present study has several limitations. The chronic pain patients investigated in this study represented one extreme of the total chronic pain patient population and should not be viewed as being representative of the Japanese chronic pain population in general. Our patients had a long history of pain. Consequently, Rorschach findings may be related to other dimensions of psychological adaptation to disease and health-related quality of life. Our study involved a female to male ratio of 2:1 and therefore psychological characteristics might be more reflective of female patients. Further studies should be designed to take this into account. In the present study, comparisons were made to normative data from non-patient Japanese adults. Future investigations have already been implemented using control data. Other types of psychological assessment measures specific to pain could be added as a test battery in order to further our understanding of the more specific features of patients with chronic pain. Interactions with patients and medical professionals were not examined in this study and future research will be needed to help the patient-doctor relationship become more productive and therapeutic.

## Conclusions

A comprehensive series of psychological characteristics determined from Japanese patients with non-malignant chronic pain were examined and compared to non-patient Japanese adults using the Rorschach CS. Results showed that these patients demonstrated high emotional distress, moderate cognitive dysfunction, and ineffective interpersonal interactions. This was in spite of the fact that they originally exhibited adequate psychological resources with capacities for control, stress tolerance and adequate interpersonal skills. In consideration with the multi-dimensional experience of pain, we recommend that medical professionals incorporate some degree of psychological intervention into conventional pain treatment regimens.

## Competing interests

The authors declare that they have no competing interests.

## Authors' contributions

KY conceptualized and designed the study, collected and analyzed the data, interpreted the results, and drafted the manuscript. KK and MF helped in drafting the manuscript. MY helped in the Rorschach analysis while AT and IB helped with the statistical analysis. HM, YM, TA and YN provided overall advice. All authors read and approved the final manuscript.
